# Necroptosis-related lncRNA in lung adenocarcinoma: A comprehensive analysis based on a prognosis model and a competing endogenous RNA network

**DOI:** 10.3389/fgene.2022.940167

**Published:** 2022-09-08

**Authors:** Fuling Mao, Zihao Li, Yongwen Li, Hua Huang, Zijian Shi, Xuanguang Li, Di Wu, Hongyu Liu, Jun Chen

**Affiliations:** ^1^ Department of Lung Cancer Surgery, Tianjin Medical University General Hospital, Tianjin, China; ^2^ Tianjin Key Laboratory of Lung Cancer Metastasis and Tumor Microenvironment, Tianjin Lung Cancer Institute, Tianjin Medical University General Hospital, Tianjin, China; ^3^ Quantitative Biomedical Research Center, Department of Population and Data Sciences, University of Texas Southwestern Medical Center, Dallas, TX, United States; ^4^ Department of Thoracic Surgery, First Affiliated Hospital, School of Medicine, Shihezi University, Shihezi, China

**Keywords:** necroptosis, lung adenocarcinoma, lncRNA, tumor microenvironment, ceRNA, cyclin dependent kinases

## Abstract

**Background:** Necroptosis, an innovative type of programmed cell death, involves the formation of necrosomes and eventually mediates necrosis. Multiple lines of evidence suggest that necroptosis plays a major role in the development of human cancer. However, the role of necroptosis in lung adenocarcinoma (LUAD) remains unclear. In this study, we aimed to construct an NRL-related prognostic model and comprehensively analyze the role of NRL in LUAD.

**Methods:** A necroptosis-related lncRNA **(**NRL) signature was constructed in the training cohort and verified in the validation and all cohorts based on The Cancer Genome Atlas database. In addition, a nomogram was developed. The tumor microenvironment (TME), checkpoint, human leukocyte antigen, and m6A methylation levels were compared between low-risk and high-risk groups. Then, we identified five truly prognostic lncRNAs (AC107021.2, AC027117.1, FAM30A, FAM83A-AS1, and MED4-AS1) and constructed a ceRNA network, and four hub genes of downstream genes were identified and analyzed using immune, pan-cancer, and survival analyses.

**Results:** The NRL signature could accurately predict the prognosis of patients with LUAD, and patients with low risk scores were identified with an obvious “hot” immune infiltration level, which was strongly associated with better prognosis. Based on the ceRNA network, we postulated that NRLs regulated the TME of patients with LUAD *via* cyclin-dependent kinase (CDK) family proteins.

**Conclusion:** We constructed an NRL signature and a ceRNA network in LUAD and found that NRLs may modulate the immune microenvironment of LUAD *via* CDK family proteins.

## 1 Introduction

According to statistics, there were 2.2 million new cases of lung cancer and 1.8 million deaths in 2020, making lung cancer the second most prevalent cancer and the leadingcause of cancer-related deaths, accounting for approximately 1 in 10 (11.4%) diagnosed cancers and 1 in 5 (18.0%) deaths (Sung et al., 2021). Worse yet, the mortality and incidence of lung cancer are increasing (Bade and Dela Cruz, 2020). Among lung cancers, lung adenocarcinoma (LUAD) is the most common subtype of non–small-cell lung cancer (NSCLC), accounting for 50% of all NSCLC cases (Denisenko et al., 2018; Peng et al., 2018). Although the widespread application of targeted therapy and immunotherapy has improved the prognosis of some patients, the survival rate remains far from satisfactory (Miller et al., 2019).

Necroptosis is an innovative type of programmed cell death that is regulated by receptor-interacting protein kinase 1 (RIPK1), RIPK3, mixed lineage kinase domain-like (MLKL), and other proteins. Caspase is well known as an essential component of apoptosis that can modulate multiple pathways (Wong, 2011; Maes et al., 2017). Only when caspase activity is inhibited can some death-related receptors, such as tumor necrosis factor receptors, be activated, resulting in the activation of downstream RIPK1, RIPK3, and MLKL and formation of necrosomes, eventually mediating necroptosis (Nunes et al., 2014; Cao et al., 2018; Yuan et al., 2019). Multiple lines of evidence suggest that the necroptotic signaling pathway plays a role in tumorigeneses, metastasis, and necrosis, eliciting immunogenicity and promoting natural or therapeutically driven anticancer immunosurveillance (Galluzzi et al., 2017; Yan et al., 2022). Although necroptosis has received increased attention, the mechanism and role of necroptosis in LUAD remain unclear.

Long non-coding RNAs (lncRNAs) are RNA molecules with nearly 200 nucleotides that regulate gene expression at the RNA splicing, transcription, and post-transcriptional levels, primarily participating in the epigenetic regulation of human tumors. Increasing evidence suggests that lncRNAs can disrupt gene expression, resulting in cancer progression (He et al., 2016; Dai et al., 2021). Furthermore, Jiang et al. (2021) hypothesized that lncRNA could control programmed cell death, such as autophagy, apoptosis, ferroptosis, and necroptosis.

Several markers have been found to predict the prognosis of patients with LUAD, but they have not been clinically used (Lin et al., 2021). Therefore, finding an optimal method in LUAD is required. This study used The Cancer Genome Atlas (TCGA) public data to perform a systematic bioinformatics analysis of necroptosis-related lncRNA (NRL) and constructed a prognostic prediction signature. Our findings may provide new evidence for predicting prognosis and therapeutic targets in patients with LUAD.

## 2 Materials and methods

### 2.1 Data collection

The transcriptome data and matching clinical information were extracted from TCGA database, which contained 497 LUAD samples and 54 adjacent normal lung tissues. The fragments per kilobase of sequence per million mapped reads value was converted into transcripts per millionand normalized. The inclusion criteria were as follows: (1) LUAD pathological type of samples, (2) availability of complete clinical information, and (3) survival durationof >20 days. Ultimately, 457 patients were enrolled for further analyses. We randomly categorized the 457 patients into a training cohort (229 patients) and a validation cohort (228 patients) at a proportion of 1:1 based on the “caret” R package. A total of 67 necroptosis-related genes (NRGs), listed in Supplementary Table S1 were retrieved from a previous study (Zhao et al., 2021). The mutation statistics of 561 patients with LUAD were acquired from TCGA database on 30 December 2021. Pan-cancer data were extracted from the Xena browser (https://xenabrowser.net/datapages/), which comprised 33 cancer types. All data used in this study are publicly available.

### 2.2 Somatic mutation and copy number variations (CNV) analysis of NRGs

The tumor mutation burden score of patients was calculatedas follows: (total mutation ÷ total covered bases) × 10^6^. The results were analyzed using the “maftools” R package. The sites on chromosomes and the frequency of mutation alterations of NRGs were analyzed using “RCircos” R language and Perl language.

### 2.3 Establishment and validation of the NRL prognostic signature

The transcriptome data of 67 NRGs were obtained using the “limma”R package and divided into mRNA and lncRNA matrices. The correlation coefficients and *p*-values of the 67 NRGs and each lncRNA were calculated using the “limma”R package, and 3643 NRLs with coefficients >0.4 and *p* < 0.00001 were selected. A false discovery rate (FDR) of 0.05 and log2|FC| of >1 were used to filter out differentially expressed NRLs. We also constructed a volcano plot. Univariate Cox regression analysis with a *p*-value of <0.05 was used to select the prognostic NRLs. The LASSO regression analysis was used to identify the prognostic NRL signature by the “glmnet” R package, and the value of the penalty parameter (λ) was determined based on the minimum partial likelihood deviation. The lambda.min is 0.03836908 and the lambda.1se is 0.1285979 in establishing signature using LASSO regression analysis. The risk scores were calculatedas follows: risk score = sum (each gene’s expression × corresponding coefficient). The corresponding coefficient of each lncRNA is shown in Supplementary Table S2. The survival, receiver operating characteristic (ROC), risk plot, t-distributed stochastic neighbor embedding (t-SNE), principal component, and subgroup analyses were performed to test the accuracy of the NRL signature.

### 2.4 Construction of nomogram and calibration

Using univariate and multivariate Cox regression, independent analysis was performed on clinical characteristics (including age, sex, clinical stage, and TNM classification of malignant tumors stage) and the NRL signature (Lu et al., 2021). Then, the nomogram and calibration diagram were developed usingthe “Regplot” R package.

### 2.5 Multifaceted analyses between high-risk and low-risk groups

#### 2.5.1 Functional enrichment analysis of differently expressed genes (DEGs)

The DEGs between the high-risk and low-risk groups were screened using the “edgeR” R package (| log2FC| of ≥1 and FDR of <0.05). Gene ontology (GO) and Kyoto Encyclopedia of Genes and Genomes (KEGG)were used to investigate the enriched pathways associated with the DEGs. Using a gene set (c2. cp.kegg.v7.4. symbols.gmt), gene set enrichment analysis (GSEA) was used to identify the significantly enriched pathways in both groups.

#### 2.5.2 Estimation of tumor microenvironment (TME) and m6A methylation

The immune cell expression and immune score were calculated in LUAD using the ESTIMATE and single-sample GSEA (ssGSEA) databases. The “ggpubr” R package was used to compare the levels of m6A methylation, human leukocyte antigen (HLA), and checkpoint expression between the high-risk and low-risk groups.

#### 2.5.3 Drug sensitivity analysis

Geeleher et al. (2014) developed a drug response prediction algorithm based on 138 drug actions in over 700 cell lines using the Cancer Genome Project database’s expression matrix. We used the “pRRophetic” R package (https://github.com/paulgeeleher/pRRophetic) to predict drug effects in the high- and low-risk groups.

### 2.6 The search for genuine prognostic lncRNAs

The “ggpubr” R package was used to analyze the correlation between signature lncRNAs and clinical stages to identify the lncRNAs that affect patient disease progression. The “survminer” R package was then used to compare the overall survival (OS) between the high-risk and low-risk groups. The “ggpubr” R package was used to analyze the differential expression of these lncRNAs in cancerous and adjacent non-tumor tissues.

### 2.7 Construction of competing endogenous RNA (ceRNA) network

We created a ceRNA network to highlight the potential role of genuine prognostic lncRNAs in LUAD and to demonstrate their relationship. We identified differentially expressed mRNAs and miRNAs in LUAD using the R package “limma.” To find targeted miRNAs of genuine lncRNAs, we used the StarBase website (http://starbase.sysu.edu.cn/). To predict the miRNA target genes, the MiRTarBase website (http://mirtarbase.cuhk.edu.cn) was used. The network diagram of 2lncRNA - 3miRNA - 120mRNA was created using Cytoscape.

### 2.8 Screening and analysis of hub genes

The protein–protein interaction (PPI) network of the STRING website identified the relationship between the downstream genes with the highest confidence (0.9). The Cytohubba software was then used to identify 15 hub genes. The ssGSEA database was used to examine the relationship between immune cells and hub genes. The Wilcox test was used to calculate the expression of the hub genes in most cancers, and the GEPIA database (http://gepia.cancer-pku.cn) was used to analyze the survival (OS and DFS) of the hub genes.

### 2.9 Statistical analysis

All statistical analyses were performed using the R and Perl languages, except descriptive analysis. All *p*-values or FDRs were calibrated using the Benjamini–Hochberg method (Ren et al., 2021). A *p*-value of <0.05 was considered statistically significant.

## 3 Results

### 3.1 Genetic variation of NRGs in LUAD

**FIGURE 1:**
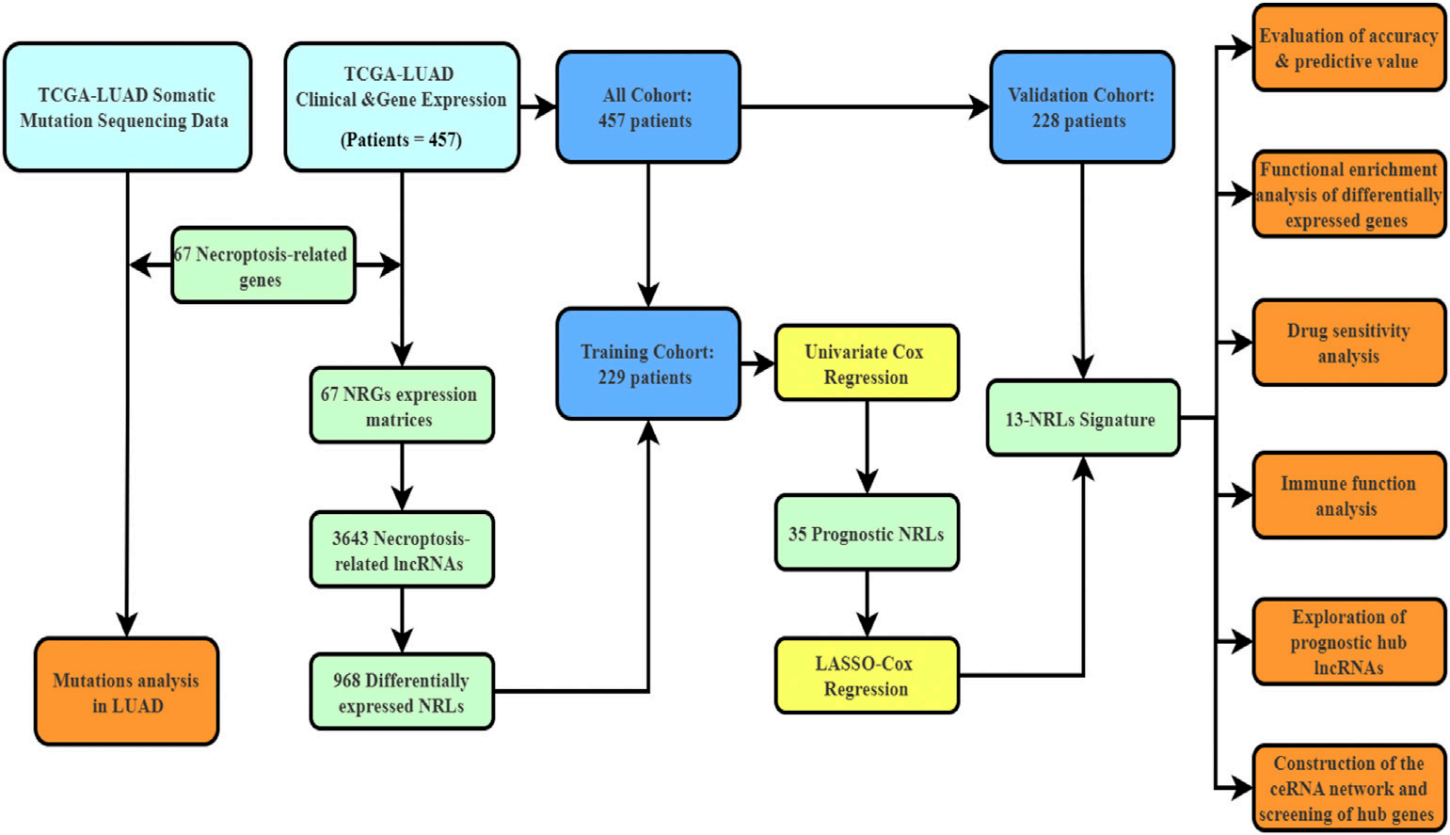
The workflow diagram of this study.

Figure 1 depicts the workflow diagram. We examined the number of somatic mutations and CNV in LUAD to investigate the NRG mutation. As shown in Supplementary Figure S1A, 296 of 561 LUAD samples (52.76%) developed NRG mutation, with missense mutation being the most common variation. In addition, the estimated glomerular filtration rate mutation frequency was highest in 67 NRGs, followed by HDAC9 and BRAF. Supplementary Figures S1B,C show the chromosome mutation sites and CNV variation frequency of these 67 NRGs.

### 3.2 Construction and validation of the NRL prognostic signature

**FIGURE 2:**
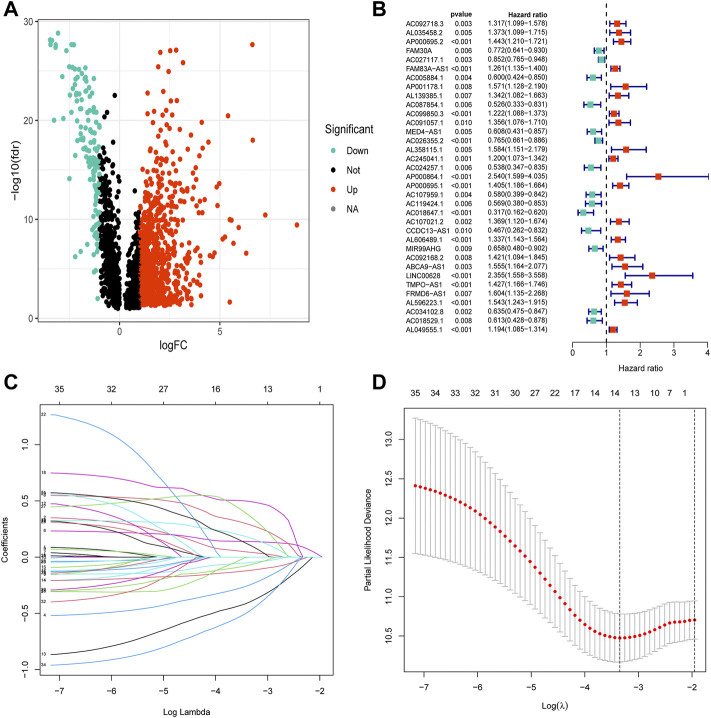
Construction of the necroptosis-related lncRNA prognostic signature. **(A)** The volcano diagram of 968 differentially expressed necroptosis-related lncRNAs. Red and blue colors represent upregulated and downregulated lncRNAs, respectively, in LUAD. **(B)** The hazard ratio (HR) and 95% confidence interval of 35 necroptosis-related lncRNAs were calculated using the univariate Cox regression analysis. The red and blue colors represent hazardous and protective factors, respectively. **(C)** The LASSO Cox analysis of necroptosis-related lncRNAs in the training cohort. The LASSO Cox coefficient of each lncRNA associated with OS is represented as a curve. **(D)** The value of the penalty parameter (λ) was determined based on the minimum partial likelihood deviationby 10-fold cross-validation.

The clinical characteristics of 457 patients with LUAD are shown in Supplementary Table S3. The correlation and differential expression analyses revealed 968 NRLs with high or low expression in LUAD (Figure 2A). The univariate Cox regression analysis revealed 35 NRLs that were significantly related to prognosis (Figure 2B, *p* < 0.01). LASSO regression analysis was also performed, and 13 lncRNAs were screened using 10-fold cross-validation (Figures 2C,D). Finally, in the training cohort, a prognostic NRL signature was identified, which included FAM30A, AC027117.1, MED4-AS1, AC026355.2, AP000864.1, AC107021.2, FRMD6-AS1, AC018529.1, AL035458.2, FAM83A-AS1, AP001178.1, AC092168.2, and AC034102.8. Patients in the validation and all cohorts were divided into high-risk and low-risk groups based on the same median risk score (0.7443). The primary characteristics and statistical analysis results of each group are presented in Supplementary Table S4.

**FIGURE 3:**
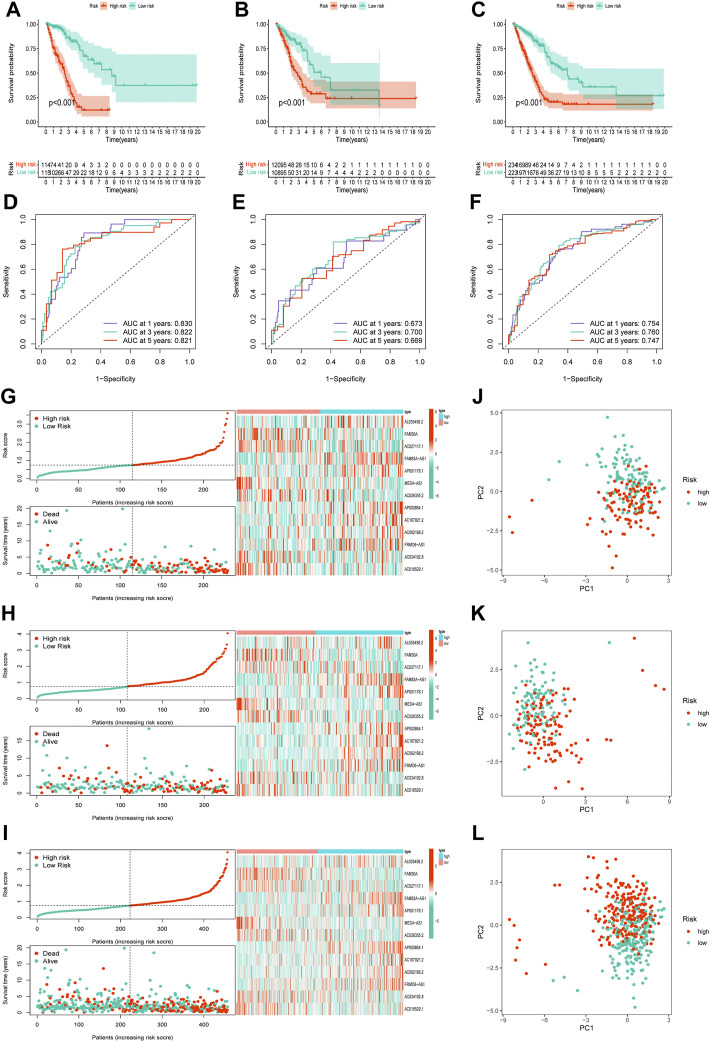
Validation of the necroptosis-related lncRNA prognostic signature. **(A–C)**The overall survival curves of high-risk and low-risk groups in the training, validation, and all cohorts. **(D–F)**The area under the curve (AUC) of the ROC curve shows the accuracy of the predictive survival signature. **(G–I)** The risk score and survival status distribution diagrams of the high-risk and low-risk groups. **(J–L)** Principal component analysis (PCA) of the high-risk and low-risk groups.

Notably, patients with LUAD with low risk scores generally had longer survival times in all three cohorts (Figures 3A–C; all *p* < 0.001). The area under the curve in the ROC curves of the training cohort was 0.830, 0.822, and 0.821, that of the validation cohort was 0.673, 0.700, and 0.669, and that of all cohort was 0.754, 0.760, and 0.747, for 1-, 3-, and 5-years OS rates, respectively (Figures 3D–F). Risk score and distributions of survival status are shown in Figures 3G–I. For all three cohorts, more deaths occurred in the high-risk group than in the low-risk group. The heatmap suggested that FAM30A, AC027117.1, MED4-AS1, AC026355.2, AC034102.8, and AC018529.1 were down-regulated, while AL035458.2, FAM83A-AS1, AP001178.1, AP000864.1, AC107021.2, AC092168.2, and FRMD6-AS1 were upregulated in the high-risk group. The PC and t-SNE analyses showed good results (Figures 3J–L and Supplementary Figure S2).

### 3.3 Clinical subgroups analysis of the NRL signature

A clinical subgroup analysis was performed, including the conventional clinicopathological characteristics age (≥65 and <65 years), sex(female and male), clinical stage (I–II and III–IV), T stage (T1–2 and T3–4), M stage (M0 and M1), and N stage (N0–1 and N2–3), which revealed that this signature is accurate in predicting the prognosis in almost all patients with LUAD (Supplementary Figure S3).

### 3.4 Independent prognostic analysis

**FIGURE 4:**
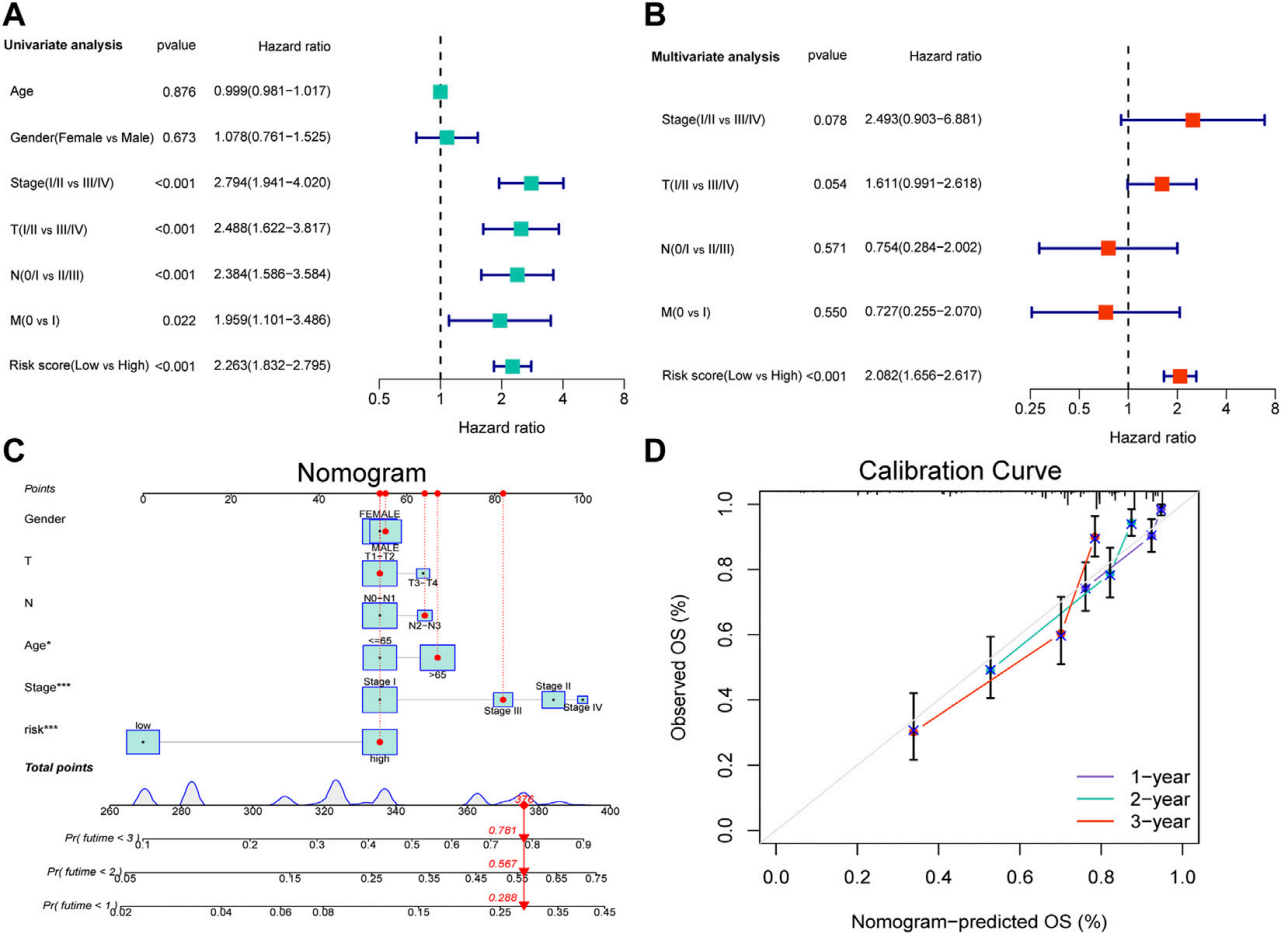
Independent prognostic analysis of the NRL signature and construction of nomogram and calibration. **(A)** The hazard ratio (HR) and 95% confidence interval of risk score and all clinical features were calculated using the univariate Cox regression analysis. **(B)**The hazard ratio (HR) and 95% confidence interval of risk score and all clinical features were calculated using the multivariate Cox regression analysis. The factor in this analysis could be considered as an independent prognostic factor when *p*-values were both <0.05 in **(A,B)**. **(C)** The nomogram that includes the risk score, age, sex, T stage, N stage, and clinical stages predicted the probability of the 1-, 2-, and 3-years OS. **(D)**The calibration curves for the predicted 1-, 2-, and 3-years OS rates.

Considering the clinicopathological features, univariate and multivariate Cox regression analyses were performed, which revealed that only risk score was an independent factor affecting the prognosis of patients with LUAD (Figures 4A,B, *p* < 0.001). In addition, a nomogram was constructed and calibration was performed, which revealed that the 1-, 2-, and 3-years OS rates could be relatively well predicted compared with an ideal model (Figures 4C,D).

### 3.5 Multifaceted analyses between high-risk and low-risk groups

#### 3.5.1 Functional enrichment analysis of DEGs

**FIGURE 5:**
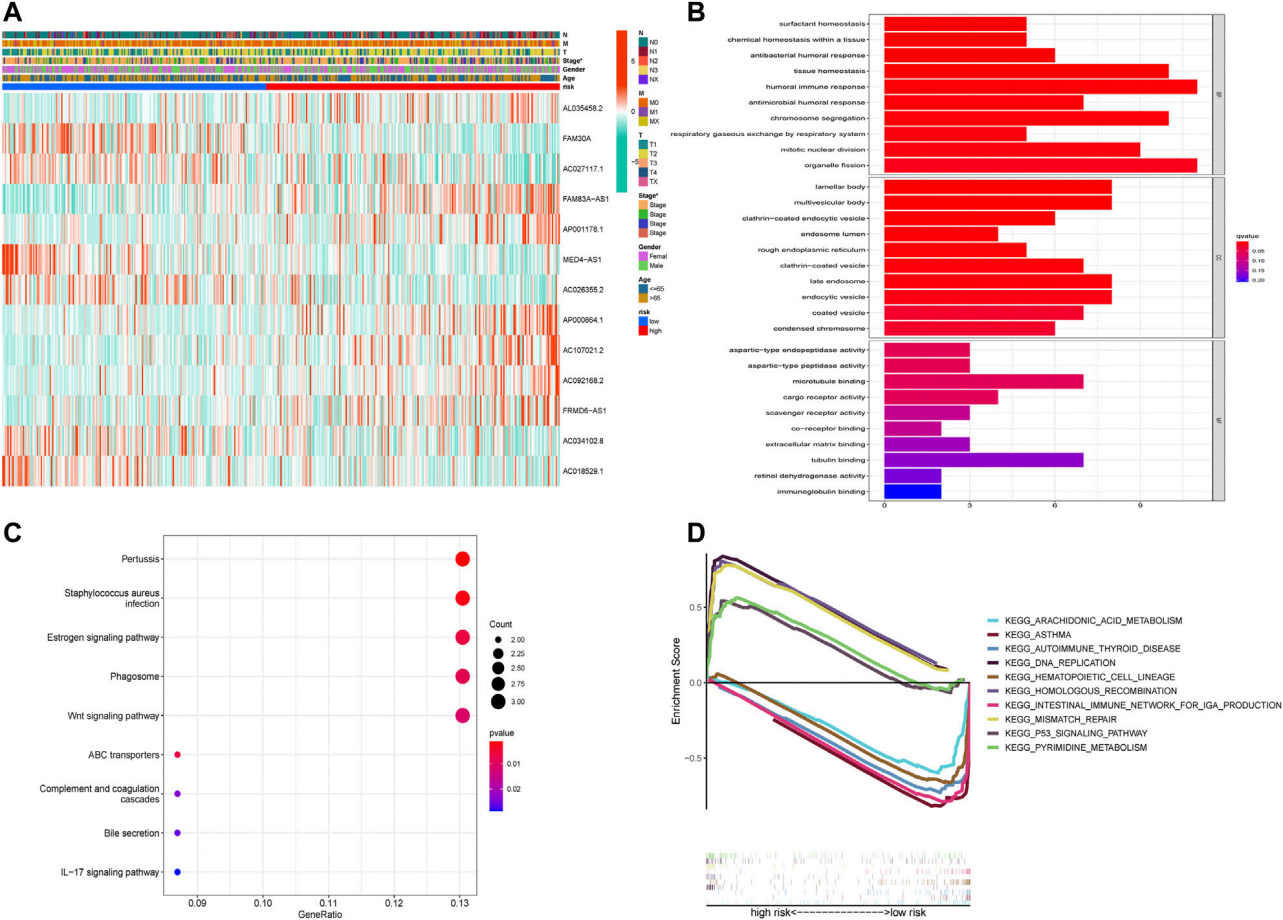
Functional enrichment analysis of DEGs in the high-risk and low-risk groups. **(A)** The heatmap of differences in clinical characteristics and lncRNA expression between the high-risk and low-risk groups. **(B)** The enriched gene terms in gene ontology (GO) analysis, including biological process, cellular component, and molecular function. The length of bars represents the number of enriched genes. **(C)** The enriched gene terms in the Kyoto Encyclopedia of Genes and Genomes (KEGG) analysis. The size of the circles represents the number of enriched genes. The horizontal axis represents the percentage of the number of genes enriched in a term to all the differentially expressed genes. The color represents statistical significance. The darker the color, the more significant the result, with red representing the most significant result. **(D)** The enriched gene terms in gene set enrichment analysis (GSEA).

A heatmap was created to further understand the differences in clinical characteristics and lncRNA expression between high-risk and low-risk groups, which revealed higher number ofstage III and IV patients in the high-risk group, while age, sex, and TNM stage were similar in both groups (Figure 5A). The GO enrichment analysis revealed that pathways were highly enriched on humoral immune response, organelle fission, tissue homeostasis, chromosome segregation, mitotic nuclear division, antimicrobial humoral response, and antibacterial humoral response (Figure 5B). KEGG analysis revealed that DEGs were mostly associated with pertussis, *Staphylococcus aureus* infection, the estrogen signaling pathway, phagosome, and the Wnt signaling pathway (Figure 5C). GSEA showed that genes were significantly enriched on DNA replication, homologous recombination, mismatch repair, pyrimidine metabolism, and the p53 signaling pathway in the high-risk group and asthma, intestinal immune network, autoimmune thyroid disease, hematopoietic cell lineage, and arachidonic acid metabolism in the low-risk group (Figure 5D).

#### 3.5.2 TME, checkpoint, HLA, and m6A methylation analysis in LUAD

**FIGURE 6:**
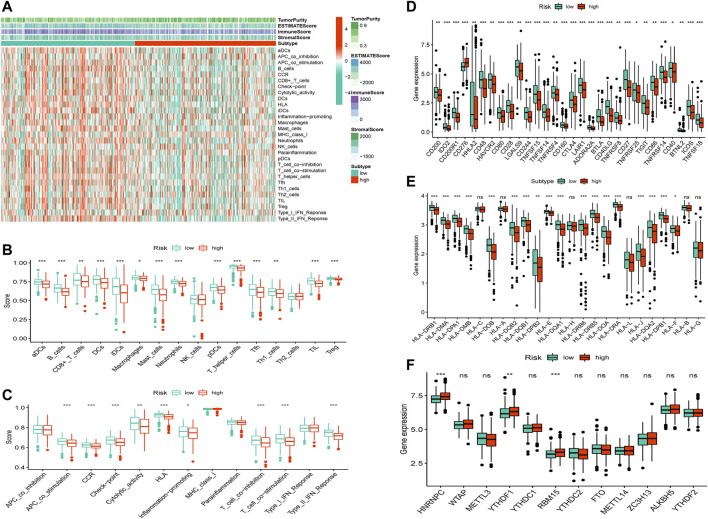
TME, checkpoint, HLA, and m6A methylation analysis in LUAD. **(A)** The heatmap of immune cells and estimate, immune, and stromal scores between the high-risk and low-risk groups. **(B)** The box plots of immune cells between the high-risk and low-risk groups. **(C)**The box plots of immune-related pathways between the high-risk and low-risk groups. **(D)** The box plots of checkpoint-related genes between the high-risk and low-risk groups. **(E)** The box plots of HLA-related genes between the high-risk and low-risk groups. **(F)** The box plots of m6A methylation-related genes between the high-risk and low-risk groups.*p < 0.05,**p < 0.01, ***p < 0.001.

The ESTIMATE and ssGSEA databases revealed that the high-risk group had lower estimate, immune, and stromal scores but higher tumor purity than the low-risk group (Figure 6A and Supplementary Figure S4A, *p* < 0.001). In addition, we determined the expression levels of infiltrating immune cells and pathways and found that the expression of aDCs, B cells, CD8^+^ T cells, DCs, iDCs, macrophages, mast cells, neutrophils, pDCs, T helper cells, Tfh, Th1 cells, TIL, and Treg were higher in the low-risk group than in the high-risk group. Moreover, the expression of co-stimulation, CCR(CC chemokine receptor), checkpoints, cytolytic activity, HLA, inflammation-promoting response, T-cell co-inhibition, T-cell co-stimulation, and Type II interferonresponse was lower in the high-risk group than in the low-risk group (Figures 6B,C). Further, the risk score was inversely associated with the number of plasma cells, dendritic cells, and monocytes but directlyassociated with the number of macrophage M0 cells, CD4^+^T cells, and CD8^+^T cells (Supplementary Figure S4A). Thus, the levels of m6A methylation and checkpoint gene and HLA expression between the high-risk and low-risk groups were evaluated, which revealed that the m6A methylation genes HNRNPC, YTHDF1, and RBM15 were significantly higher in the low-risk group than in the high-risk group, but other m6A methylation genes did not differ significantly between the groups. Remarkably, most checkpoint genes were significantly highly expressed in the low-risk group (Figures 6D–F).

#### 3.5.3 Drug sensitivity analysis

The “pRRophetic” R package was used to predict drug effects in patients with LUAD based on the drug response prediction formula described by Geeleher et al. The outcomes and drug details are presented in Supplementary Figure S5 and Supplementary Table S5, respectively. We found that A.443654, cisplatin, CGP.60474, docetaxel, epothilone B, GW843682X, NVP-TAE684, and paclitaxel showed high sensitivity in the low-risk group, while ABT.888, bosutinib, lenalidomide, MK.2206, PAC.1, PD.0332991, and roscovitine showed high sensitivity in the high-risk group.

### 3.6 The search for genuine prognostic lncRNAs

We believe that lncRNA, which is closely related to tumor progression, is the genuine prognostic lncRNA. To identify these genuine lncRNAs, the correlation between the signature lncRNAs and clinical stages was evaluated (Supplementary Figure S6). Finally, five lncRNAs, including AC107021.2, AC027117.1, FAM30A, FAM83A-AS1, and MED4-AS1, were identified. The survival curve and differential expression of these lncRNAs were analyzed (Supplementary Figure S7). Ultimately, AC027117.1 and FAM30A were used to construct a ceRNA network.

### 3.7 Construction of ceRNA network

**FIGURE 7:**
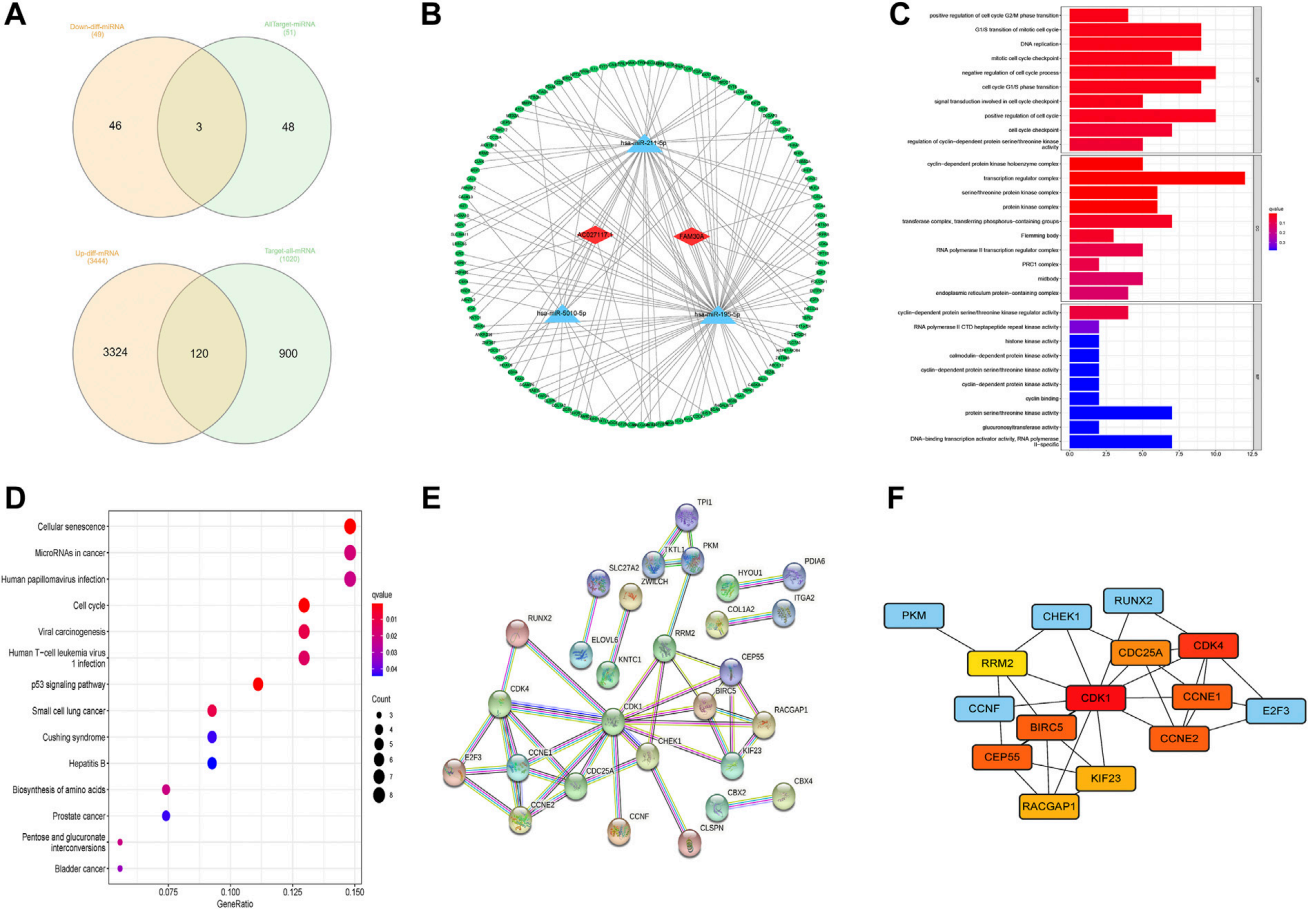
Construction of competing endogenous RNA network. **(A)** The predicted results of targeted miRNAs and targeted mRNAs obtained using StarBase and MiRTarBase. **(B)** The competing endogenous RNA (ceRNA) network between 2 lncRNAs, 3 miRNAs, and 120 downstream mRNAs. **(C)** The enriched terms of downstream genes in gene ontology (GO) analysis. **(D)** The enriched terms of downstream genes in the Kyoto Encyclopedia of Genes and Genomes (KEGG) analysis. **(E,F)** A protein–protein interaction (PPI) analysis of downstream genes and network of hub genes.

LncRNA has been shown to be involved in controlling the expression of genes as a miRNA sponge(Salmena et al., 2011; Karagkouni et al., 2021). The present study aimed to discover a network of lncRNA, miRNA, and mRNA. First, we used the StarBase website (http://starbase.sysu.edu.cn/) to predict the targeted miRNAs of AC027117.1 and FAM30A and obtained 51 targeted miRNAs. The differentially expressed miRNAs and mRNAs in LUAD were explored, and eventually, differentially expressed 617 miRNAs and 14,147 mRNAs were identified(|logFC> 1|, *p* < 0.05). Then, 49 downregulated differentially expressed miRNAs and 51 targeted miRNAs were overlapped, and 3 overlapping miRNAs (hsa-miR-195-5p, hsa-miR-211-5p, and hsa-miR-5010-5p) were obtained. Based on the 3 miRNAs, 120 overlapping genes were identified using the MiRTarBase website (http://mirtarbase.cuhk.edu.cn/) to predict the miRNA target genes and overlap with 3,444 upregulated differentially expressed mRNAs (Figure 7A). Cytoscape was used to draw the network to reveal the relationships between these small RNAs (Figure 7B). Finally,a ceRNA network was constructed comprising 2 NRLs, 3 miRNAs, and 120 mRNAs.

Further, we performed GO and KEGG enrichment analyses to elucidate the roles of 120 downstream genes. No significant functional, cellular, and behavioral pathways were obtained in GO analysis (Figure 7C), while KEGG genes were mainly enriched in cell senescence, microRNAs in cancer, infection, and cell cycle pathways (Figure 7D).

### 3.8 Screening and analysis of hub genes

**FIGURE 8:**
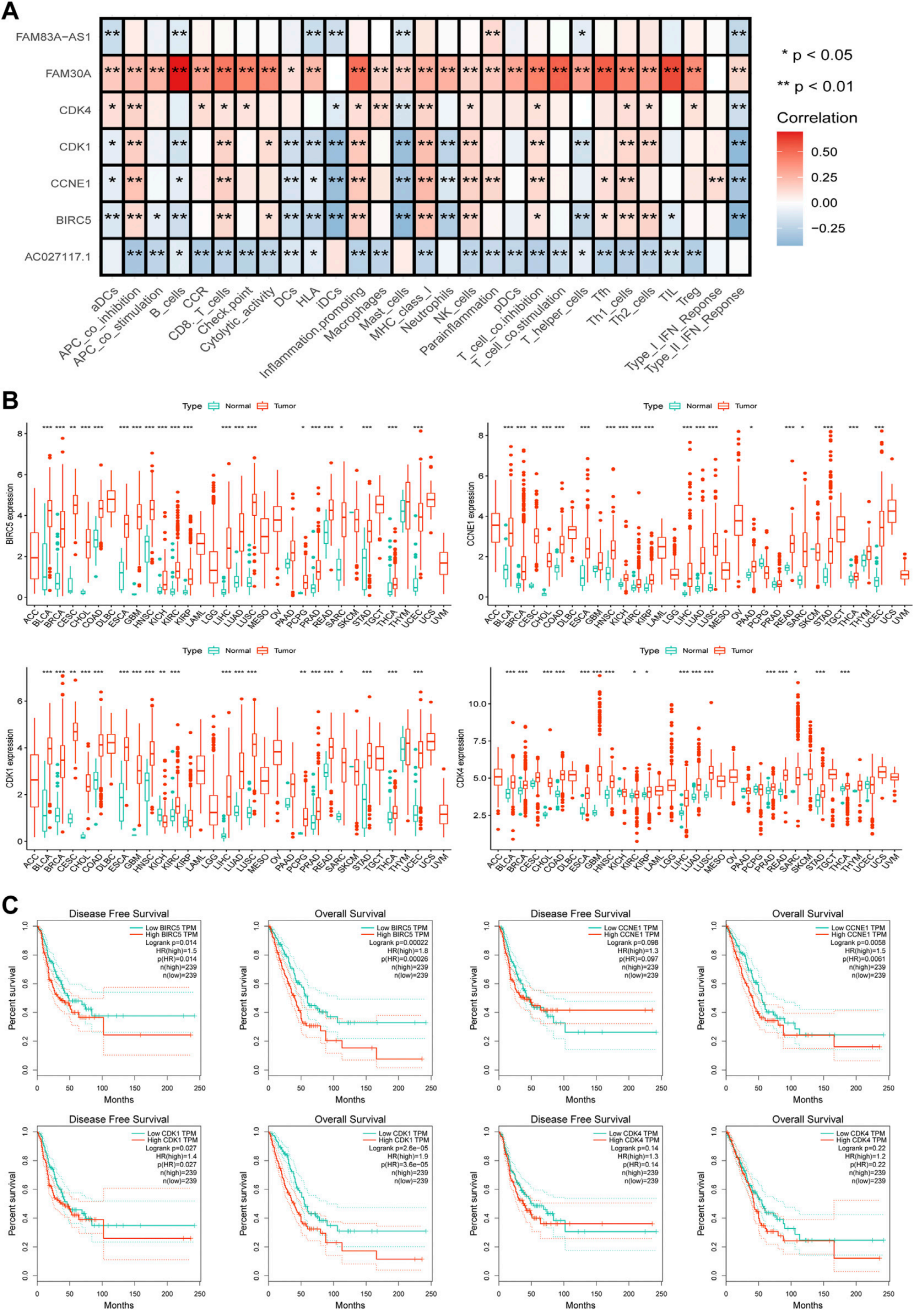
Further analysis of hub genes. **(A)** The correlation analysis between the hub genes and lncRNAs and immune functions in LUAD. **(B)** The pan-cancer analysis of four hub genes in TCGA. **(C)** The disease-free survival (DFS) and overall survival (OS) analysis curves of the four hub genes in LUAD.

Through the STRING website, a PPI network was constructed with a threshold value of 0.9, which revealed that CDK1, CDK4, CCNE1, and BIRC5 were the hub genes (Figures 7E,F). The ssGSEA database evaluation revealed that FAM30A was favorably associated with general immune cells, whereas AC027117.1 was negatively correlated with general immune cells (Figure 8A).

To further understand the roles of the four hub genes in human cancers, we performed a pan-cancer examination in 33 common cancers (Figure 8B). The four genes were found to be generally overexpressed in various cancer tissues, although some cancers did not exhibit sufficient adjacent normal tissues for statistical comparison.

The curves of disease-free survival (DFS) and OS of four genes in LUAD were analyzed using the GEPIA database, which revealed that CDK1 and BIRC5 are significantly correlated with the prognosis of patients with LUAD, and patients with high expression levels of CDK1 and BIRC5 showed poor prognoses. CDK4 was not correlated with prognosis, and CCNE1 was positively correlated with OS but not with DFS (Figure 8C).

## 4 Discussion

lncRNAs are a type of non-coding RNA consisting of approximately 200 nucleotides (Wang et al., 2021a). Increasing evidence suggests that LUAD lncRNA plays an important role in prognosis and immunotherapeutic response. Several studies have developed lncRNA prognosis signatures, such as m6A-related (Wang et al., 2021b), ferroptosis-related (Fei et al., 2021), pyroptosis-related (Song et al., 2021), immune-related (Qi et al., 2021), and metastasis-related lncRNA signatures (Dou et al., 2021). However, NRLs are still being investigated, and their prognostic role in LUAD remains unclear. Thus, we investigated NRLs systematically and developed a signature as a potential biomarker for predicting patient prognosis in LUAD.

We examined the NRGs in LUAD and found that more than half of them were mutated. We constructed a prognostic NRL signature using univariate Cox regression and LASSO regression analyses to accurately predict the prognosis of patients with LUAD, and the signature’s accuracy was confirmed using KM survival, ROC, PCA, univariate and multivariate Cox regression, and t-SNE analyses. Among the 13 lncRNAs studied, FAM30A, FAM83A-AS1, MED4-AS1, AC026355.2, and AC092168.2 were identified in previous studies, and the other lncRNAs were discovered in this study. FAM30A has previously been shown to inhibit the proliferation, invasion, and migration of laryngeal squamous cell carcinoma cells *in vitro* (Long et al., 2021). In contrast, FAM30A expression was increased in gastric cancer (GC) cell lines, and patients with GC who had high FAM30A expression had poor survival outcomes (Wang et al., 2021c). Our findings showed that FAM30A is highly expressed in low-risk patients, implying that it may hasten a good prognosis in LUAD. FAM30A was found to be differentially upregulated in periodontitis and to be positively associated with the proportion of plasma cells (Wu et al., 2020). FAM30A was identified in LUAD as a lncRNA associated with immune (Wu et al., 2021). A study of vaccine-induced immune-associated lncRNAs discovered that FAM30A was highly expressed in B cells and was closely related to immunoglobulin genes located near B cell-related genes(de Lima et al., 2019). Meanwhile, FAM30A has been found to play an important role in rheumatoid arthritis (Li et al., 2020). Surprisingly, the same result was found in our study, that FAM30A was associated with a plethora of immune and inflammatory cells, among which it was significantly positively correlated with B cells, and we speculated that FAM30A may achieve tumor-suppressive effects in LUAD by regulating the immune microenvironment by B cells. We hope that the role of FAM30A in cancer and the immune microenvironment will be investigated further in the future. Previous studies have shown that FAM83A-AS1 can sponge various miRNAs to aid progression in esophageal cell squamous carcinoma, esophageal cancer, and LUAD (Huang et al., 2020; Jia et al., 2021; Huang et al., 2022). FAM83A-AS1 was recently found to be involved in the progression of LUAD tumors (Chen et al., 2022). The antigen presentation process was found to be negatively correlated with FAM83A-AS1. Furthermore, FAM83A-AS1 was used to create signatures of ferroptosis- and pyroptosis-related lncRNA in LUAD (Guo et al., 2021; Song et al., 2021). Therefore, we believe that FAM83A-AS1 is involved in ferroptosis, pyroptosis, and necroptosis. A previous study found that MED4-AS1 was upregulated in A549 cells (human LUAD cell line) (Wang et al., 2019). However, MED4-AS1 was found to be a protective factor in our study and should be expressed at a low level in cancer cells. Furthermore, we found that high expression of AC026355.2 predicts a better prognosis in patients(He et al., 2021) and low expression of AC092168.2 predicts better outcomes (Wu et al., 2021).

We performed GO, KEGG, and GSEA analyses to determine the reasons for the differences in prognosis. We found that genes were mainly enriched in DNA repair, cell circulation, and immune, metabolic, and carcinogenic pathways. The tumor immune infiltration analysis revealed a greater number of immune cells in the low-risk group, indicating that the “hot” immune cell infiltration represents a favorable prognosis. In addition, we revealed that checkpoint-related and HLA-related genes were highly expressed in the low-risk group. These results suggest a dynamic balance between tumor and immune microenvironments. Patients will demonstrate good prognosis if the level of “hot” immune cell infiltration is higher than the level of immune checkpoint blocking. In the nomogram, we found a novel phenomenon: patients with stage 2 had a higher riskscores than patients with stage 3. This is different from our usual understanding of tumor clinical stages. We suspect that this phenomenon is more likely to be related to the differences in clinical information of patients in the TCGA database, and the reasons are still unclear.

We constructed a ceRNA network and identified four hub genes based on the five lncRNAs that may affect prognosis, including AC027117.1, AC107021.2, FAM30A, FAM83A-AS1, and MED4-AS1(CDK1, CDK4, CCNE1, and BIRC5). Later, we performed a pan-cancer analysis of the four hub genes and found that they were all overexpressed in human tumors. Differences in CDK activity can frequently result in tumor-associated cell cycle defects. CDK dysfunction causes abnormal proliferation as well as genomic and chromosomal instability (Malumbres and Barbacid, 2009). Huang et al. demonstrated that inhibiting CDK1/2/5 can mediate immune cell death and block immune checkpoint expression in pancreatic cancer, which can transform immune cell infiltration of cancers from low to high levels *via* these two mechanisms, overcoming the immune tolerance induced by interferon therapy (Huang et al., 2021). CDK4 and CDK6, as well as their activators (D-type cyclins), are thought to be a driving force in tumorigenesis and may be useful therapeutic targets (Puyol et al., 2010; Fassl et al., 2022). Furthermore, CCNE1 (Cyclin E1) is related to the CDK family. Therefore, we concluded that NRLs, CDKs, the immune microenvironment, and tumor cells are all strongly regulated.

Several models are currently being developed to predict patient prognosis in LUAD. In comparison, our model shows good stability and wide coverage in the subgroup analysis. The prediction accuracy of our model is worthy of peer review. Our model, one of the few based on necroptosis, is a novel apoptosis mechanism with a promising role in cancer. Furthermore, we not only built predictive models but also investigated the causes of necroptosis in patients with LUAD using a ceRNA network. However, we acknowledge that our study has some limitations. First, our study is based on publicly available data, with no experimental data to back it up. Second, our investigation of the role of NRLs in antitumor immunity was limited to a cursory examination of its potential mechanism. Third, our signature has only been verified internally, not externally.

## 5 Conclusions

We constructed an NRL signature and tested its accuracy in predicting the prognosis of patients with LUAD. Furthermore, we constructed a ceRNA network and hypothesized that NRLs might modulate the immune microenvironment of LUAD via CDK family proteins, influencing patient prognosis. However, these findings are only the beginning of research in this area, not the end. Thus, additional research is warranted to confirm these findings.

## Data Availability

All raw data used in this study can be downloaded from TCGA database. Details of the R code can be obtained from the corresponding author upon reasonable request.
