# Supplementary figures and images for: Necroptosis-related lncRNA in lung adenocarcinoma: A comprehensive analysis based on a prognosis model and a competing endogenous RNA network
Source: Front Genet. 2022 Sep 8;13:940167. doi: 10.3389/fgene.2022.940167 (PMC9493131; doi:10.3389/fgene.2022.940167)

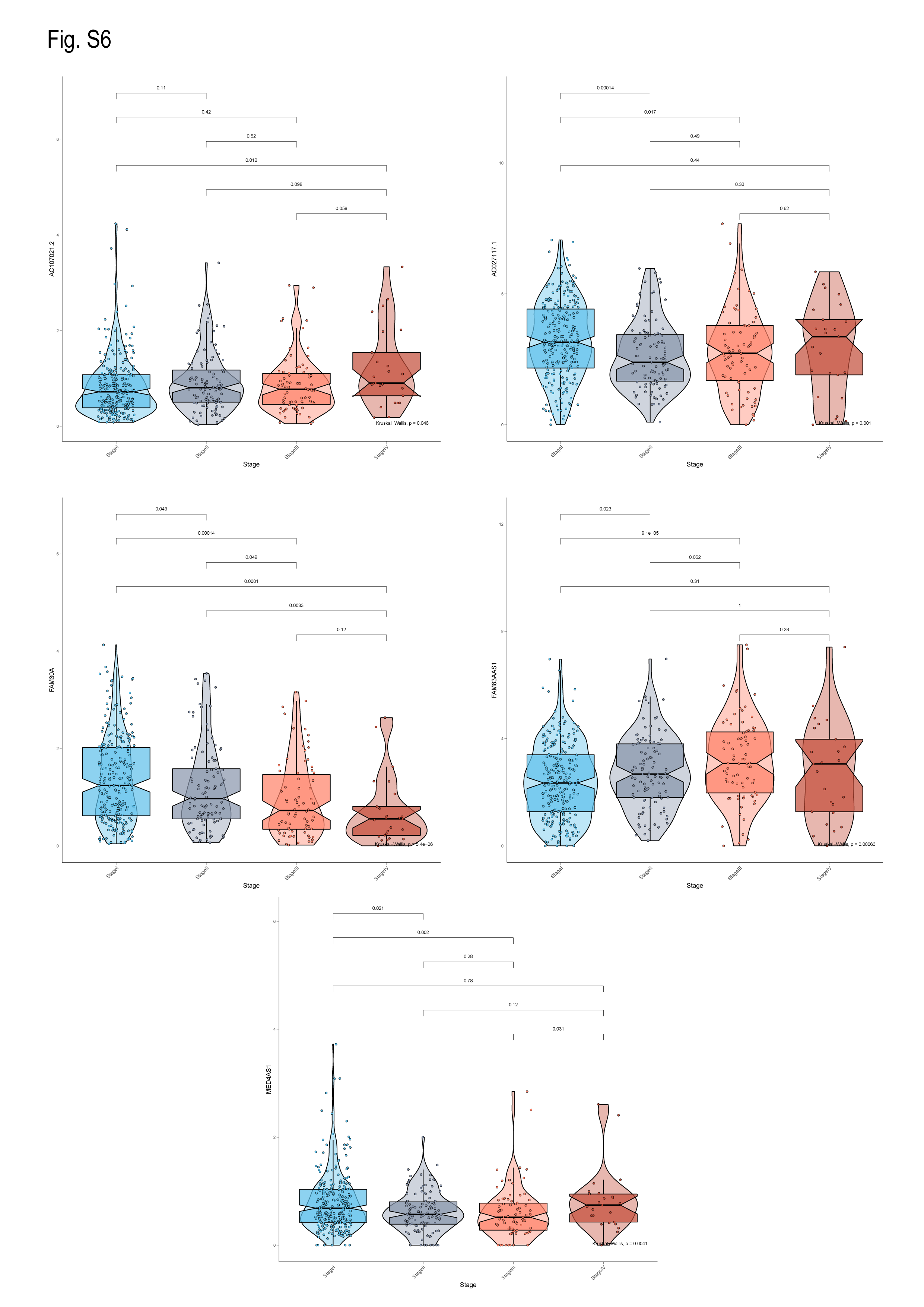

Supplement: Supplementary file 3 [file Image6.tif]

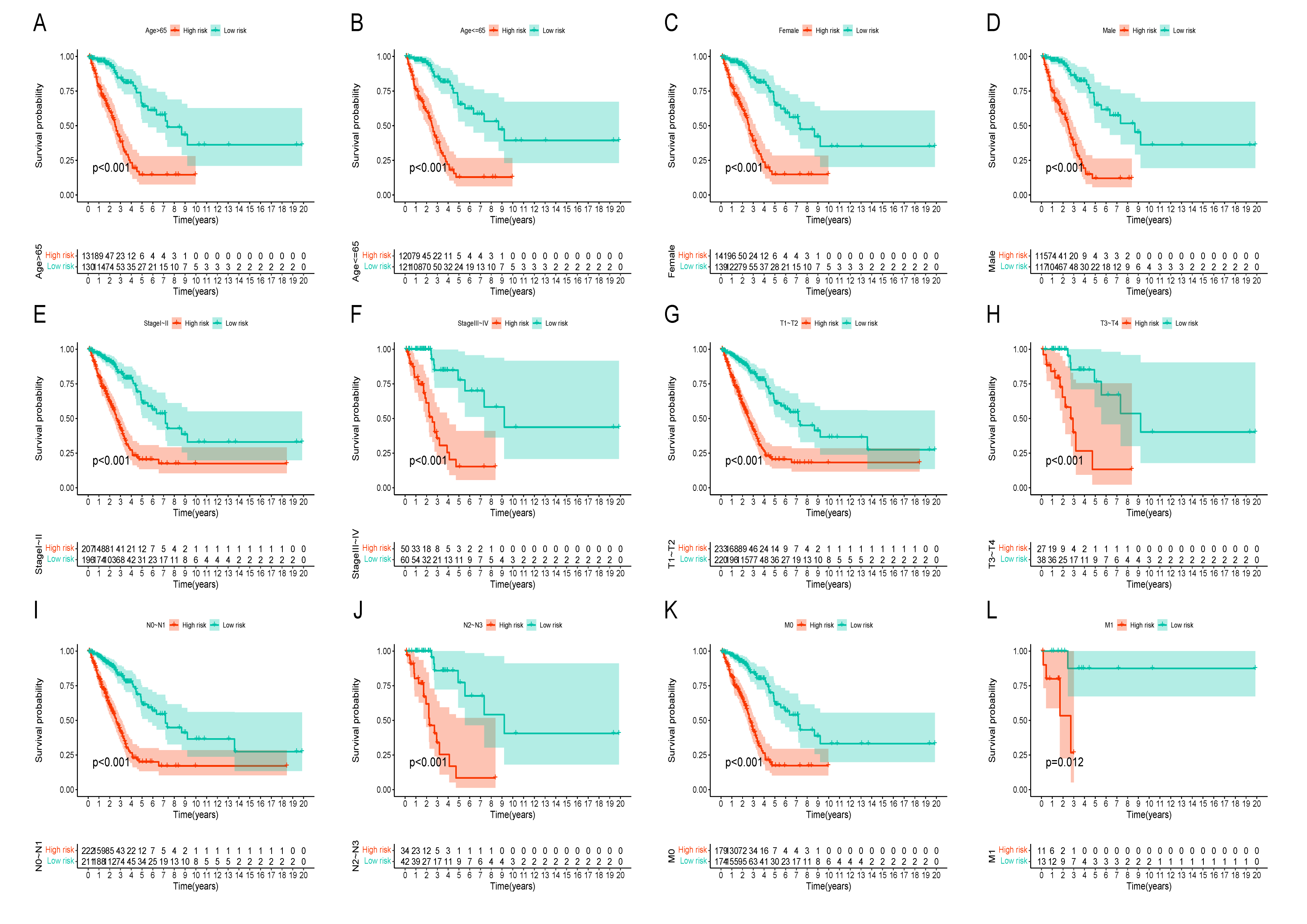

Supplement: Supplementary file 4 [file Image3.tif]

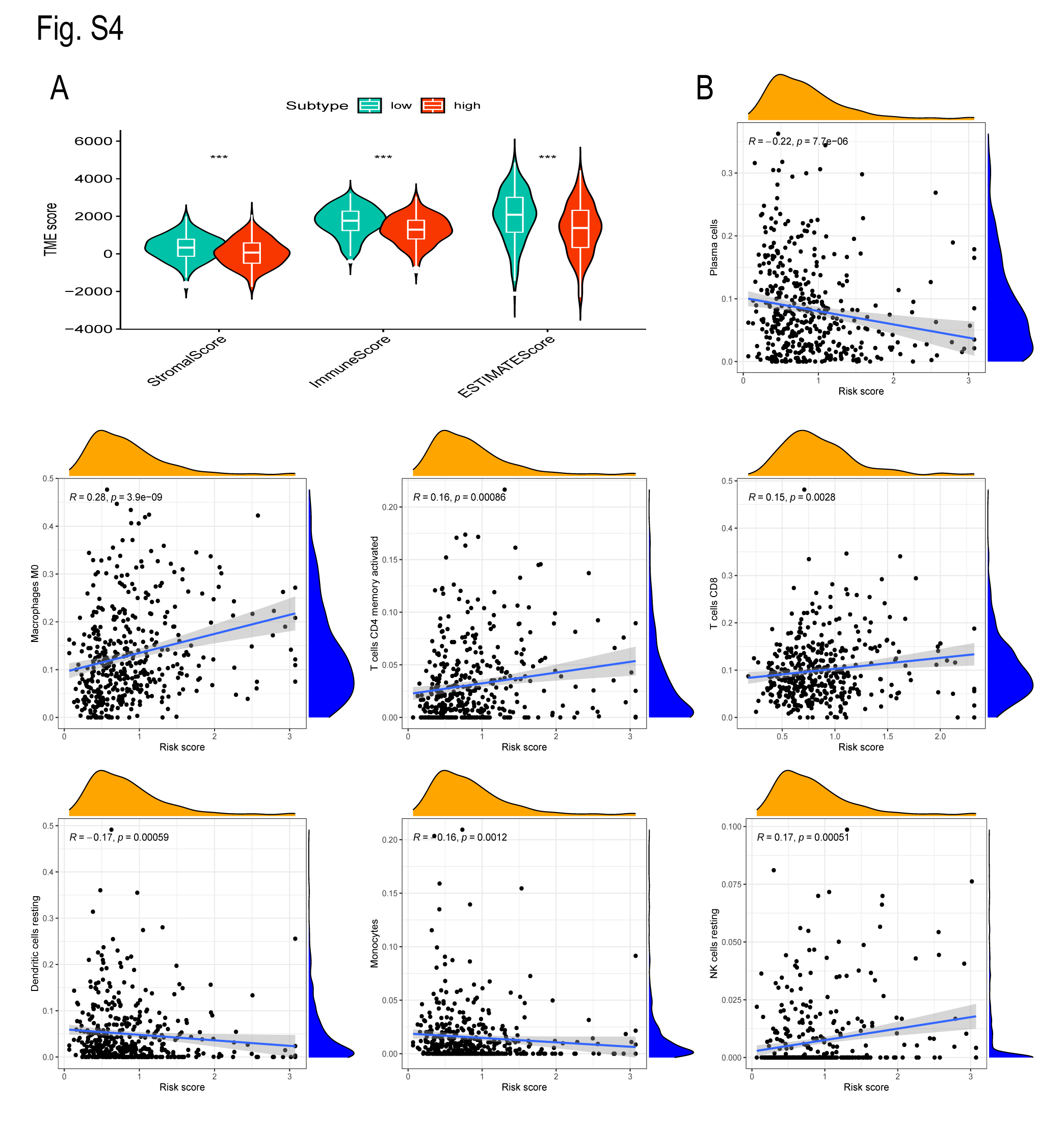

Supplement: Supplementary file 5 [file Image4.tif]

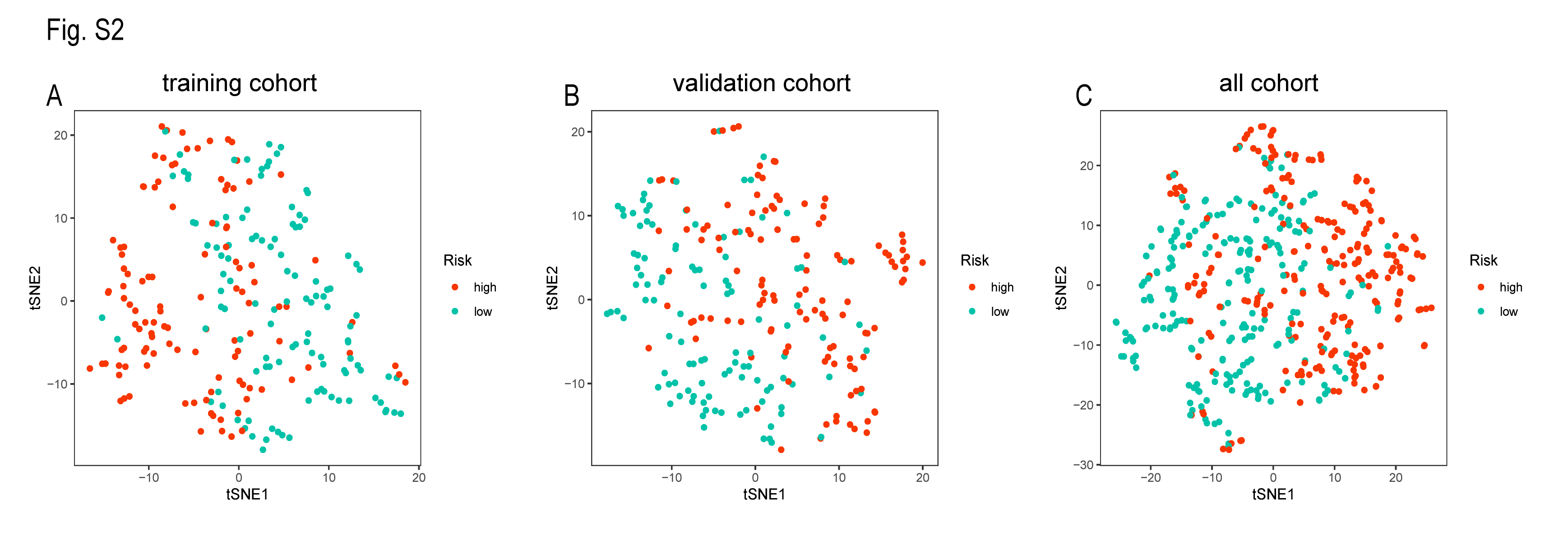

Supplement: Supplementary file 6 [file Image2.tif]

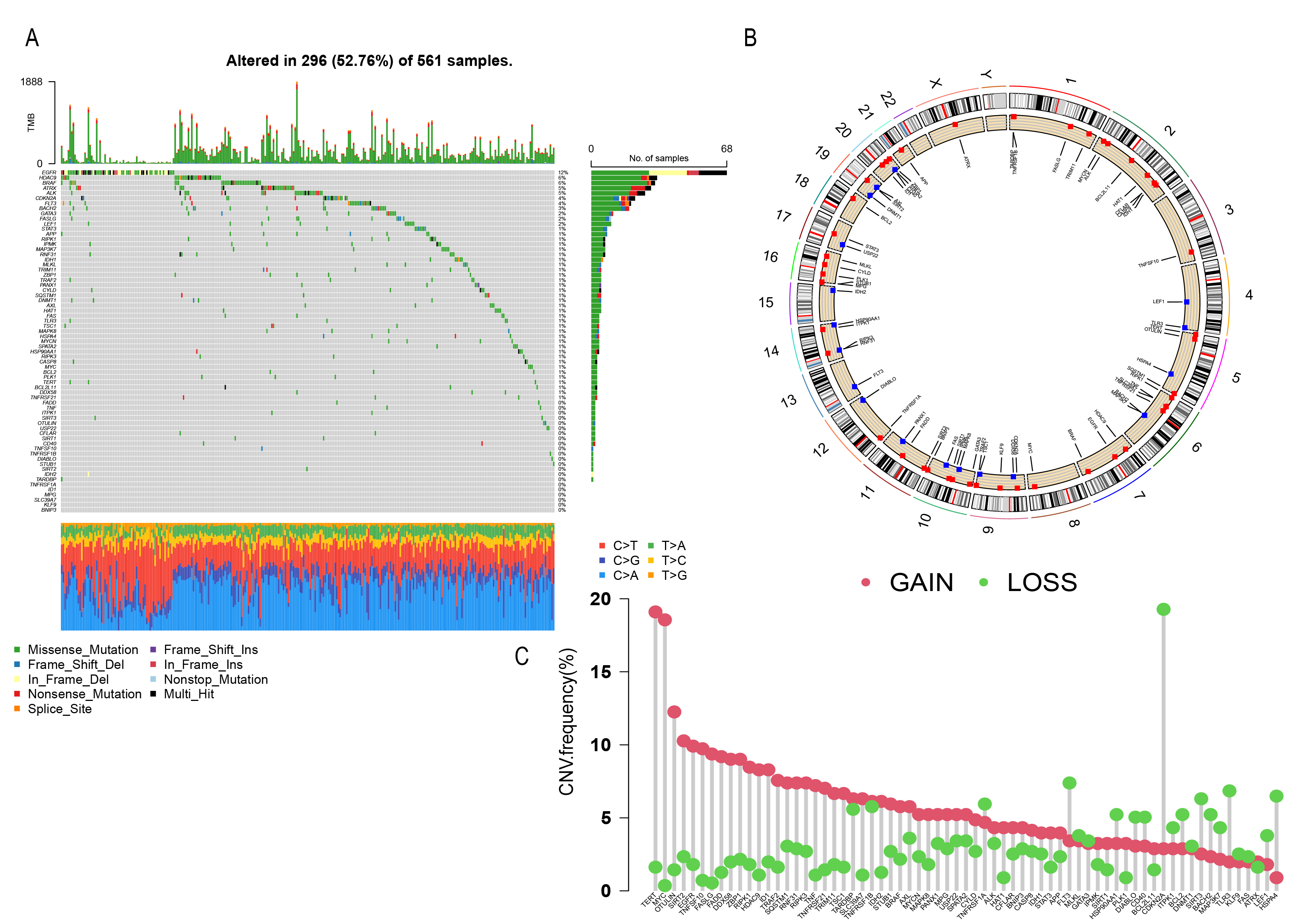

Supplement: Supplementary file 7 [file Image1.tif]

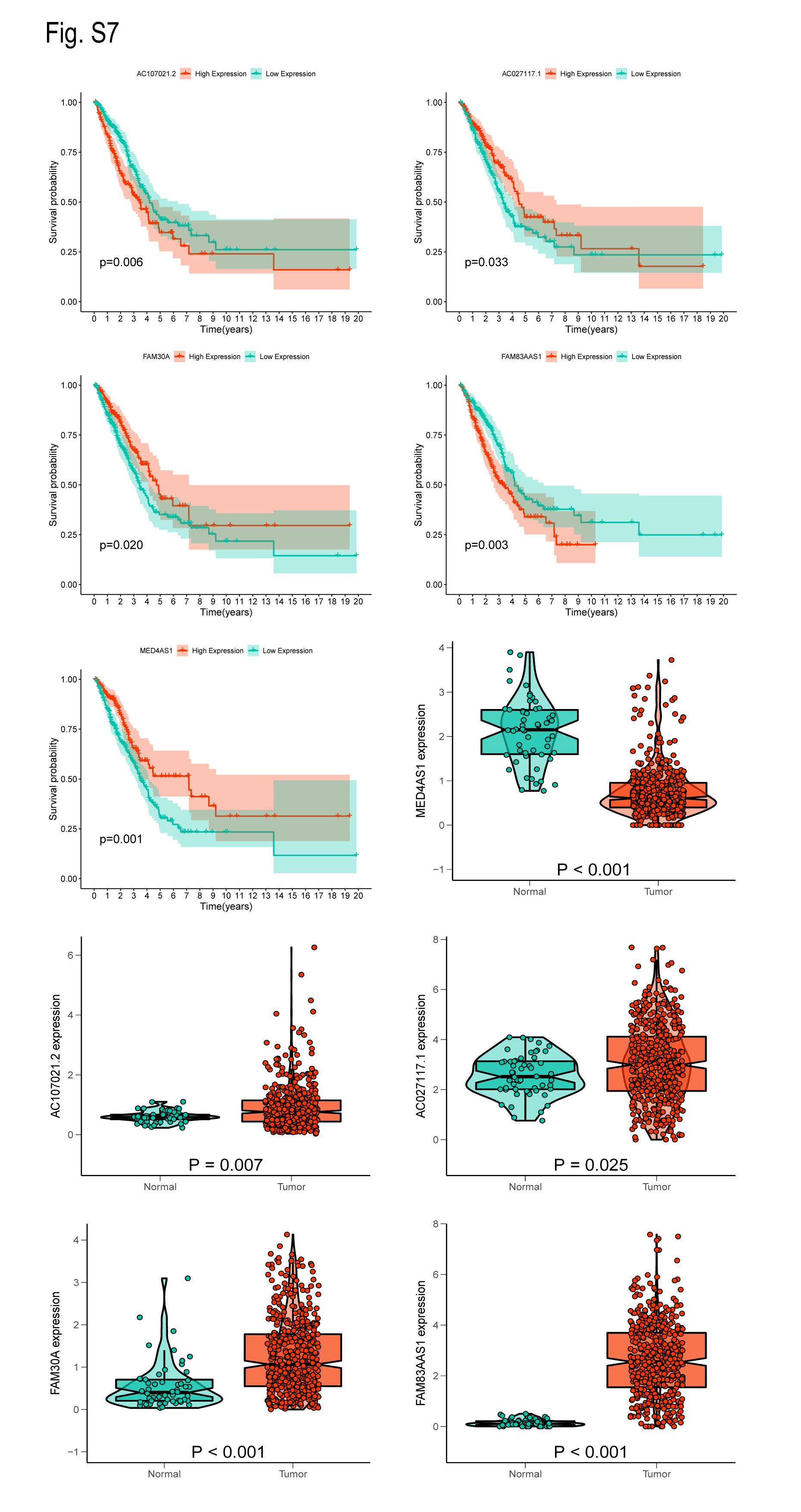

Supplement: Supplementary file 8 [file Image7.tif]

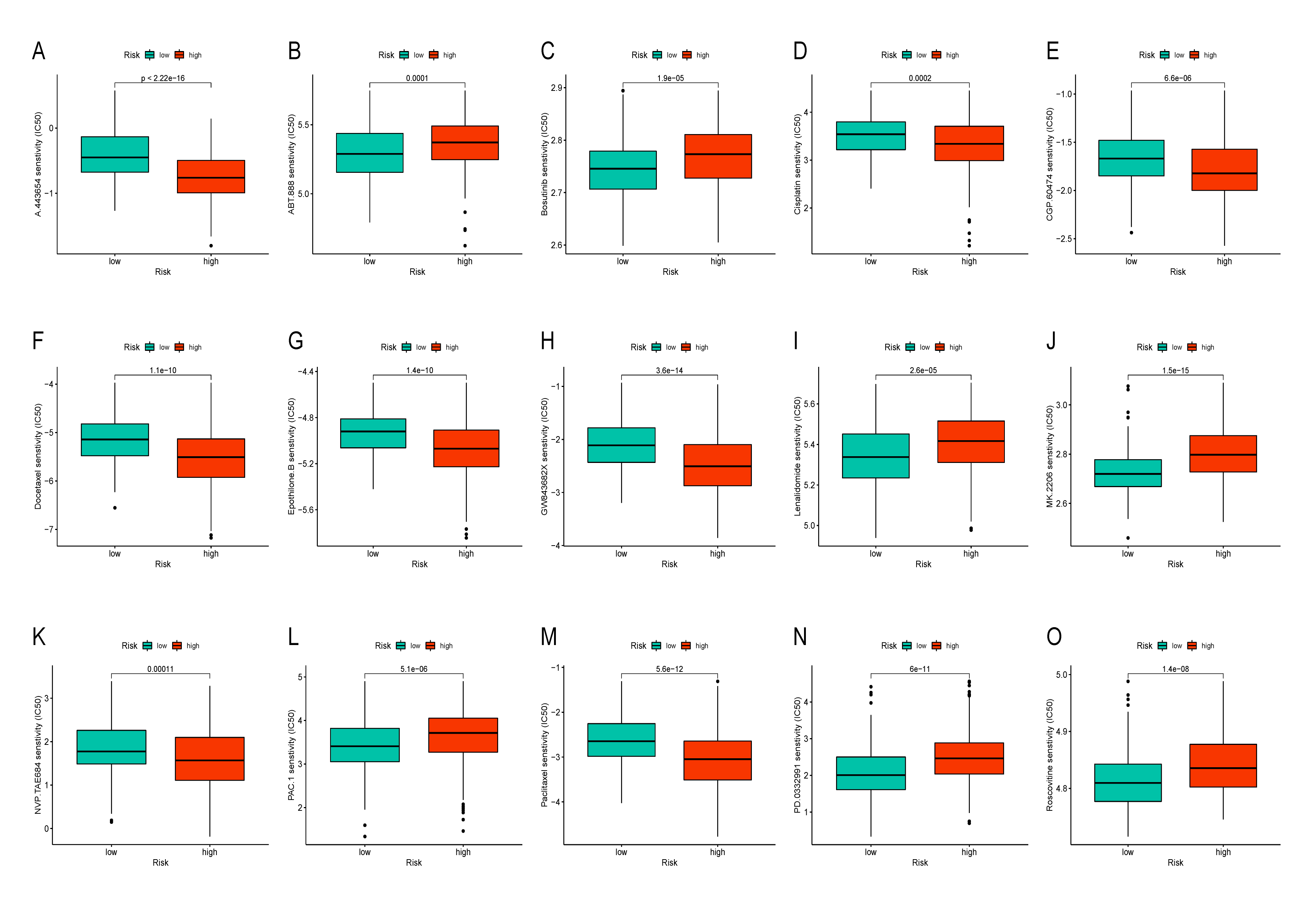

Supplement: Supplementary file 12 [file Image5.tif]
