# Supplementary material for: Necroptosis-related lncRNA in lung adenocarcinoma: A comprehensive analysis based on a prognosis model and a competing endogenous RNA network
Source: Front Genet. 2022 Sep 8;13:940167. doi: 10.3389/fgene.2022.940167 (PMC9493131; doi:10.3389/fgene.2022.940167)
Supplement: Supplementary file 11 [file Table6.docx]

**Figure S1. Landscape of genetic and expression variation of NRG in LUAD.** (A)The mutation frequency and classification of 67 NRGs in LUAD. (B) The location of gene alteration of 33 NRGs on 67 chromosomes in the LUAD. (C) The CNV variation frequency of these 67 NRGs.

**Figure S2.** The t-SNE analysis of training, validation, and all cohorts.

**Figure S3. Clinical subgroups analysis of the Signature.** Based on different clinical information, we divided patients into different subgroups and analyzed the prognostic ability of the signature, respectively.

**Figure S4. The relationship between immune microenvironment and risk score in LUAD.** (A)The comparison of estimate score, immune score, and stromal score between high-risk and low-risk groups. (B) The correlation analysis between the risk score and immune cells.

**Figure S5.** Drug sensitivity analysis of high-risk and low-risk groups.

**Figure S6.** Correlation analysis between signature lncRNAs and clinical stages.

**Figure S7.** Survival and differential expression analysis of genuine prognostic lncRNAs.

**Table S1.** Details of necroptosis-related genes.

**Table S2.** Details of the corresponding coefficient of signature lncRNAs.

**Table S3.** Clinical characteristics of LUAD patients in TCGA.

**Table S4.** Clinical characteristics of low-risk and high-risk groups.

**Table S5.** Details of the drug information.
